# ^**123**^I-MIBG Scintigraphy as a Powerful Tool to Plan an Implantable Cardioverter Defibrillator and to Assess Cardiac Resynchronization Therapy in Heart Failure Patients

**DOI:** 10.1155/2012/690468

**Published:** 2012-09-26

**Authors:** Antonella Stefanelli, Giorgio Treglia, Alessandro Giordano

**Affiliations:** Department of Radiological Sciences, Institute of Nuclear Medicine, Università Cattolica del Sacro Cuore, Largo Agostino Gemelli 8, 00168 Rome, Italy

## Abstract

Iodine-123-metaiodobenzylguanidine (^123^I-MIBG) scintigraphy is a nuclear medicine technique which describes the functional status of the cardiac sympathetic nervous system. It is well known that an autonomic dysfunction is present in heart failure setting as a neuronal uptake of norepinephrine is impaired in the failing myocardium. Reduction in sympathetic nervous function in the heart, measured by reduced myocardial uptake of ^123^I-MIBG, is an indicator of poor prognosis for heart failure patients. The aim of this paper was to investigate the role of ^123^I-MIBG scintigraphy in evaluating the need of implantable cardioverter defibrillator (ICD) and the response to cardiac resynchronization therapy (CRT) in heart failure patients. For this purpose scientific literature data on these topics were reviewed. Based on literature data, ^123^I-MIBG scintigraphy seems to be a useful tool to assess which patients may benefit most from an ICD implantation to reduce the risk of ventricular arrhythmia or sudden cardiac death. Furthermore, ^123^I-MIBG scintigraphy seems to predict which patients will response to CRT with an improvement in left ventricular function.

## 1. Introduction

Heart failure (HF) is characterized by alterations in myocardial sympathetic nerve activity; an increased sympathetic response is initially favorable by serving as compensation for decreased cardiac output, but as HF progresses, this response leads to deleterious neurohormonal and myocardial structural changes that worsen the condition and increase the likelihood of arrhythmias and cardiac death [1].

Myocardial innervation imaging with iodine 123 metaiodobenzylguanidine (^123^I-MIBG) scintigraphy provides a noninvasive tool for the investigation of cardiac sympathetic innervation; this technique can also demonstrate drug-induced changes in cardiac adrenergic activity [2].

Radiolabeled MIBG is considered an established sympathetic neuron-imaging agent useful to study the organs richly innervated by the sympathetic nervous system. MIBG is an analog of guanethidine and is taken up by the postganglionic presynaptic nerve endings of the adrenergic nervous system [3–6]. After depolarization, MIBG is released into the synaptic cleft like norepinephrine but is not metabolized. Labeling MIBG with ^123^I allows the visualization of adrenergic innervation in vivo; ^123^I-MIBG scintigraphy not only displays the presence of noradrenergic innervation but also its functional capability [3–6].

About the scintigraphic procedure, planar images of the thorax are acquired 15 minutes (early image) and 4 hours (delayed image) after radiopharmaceutical injection with the patient lying in the same supine position. Tomographic (SPECT) images are usually performed after early and delayed planar imaging [3].

^123^I-MIBG uptake is semiquantified by calculating the heart to mediastinum ratio (H/M), after drawing regions of interest (ROI) over the heart and the upper mediastinum in the planar anterior view. Average counts per pixel in the myocardium are divided by average counts per pixel in the mediastinum [3–6]. The myocardial washout rate (WR) from initial to late images is also calculated, and expressed as a percentage, as the rate of decrease in myocardial counts over time between early and late imaging [3–6].

The delayed H/M ratio reflects the relative distribution of sympathetic nerve terminals, offering global information about neuronal function resulting from uptake, storage, and release. The WR reflects the neuronal integrity or sympathetic tone [3]. Impaired sympathetic activity in HF is usually associated with high myocardial WR and low early and delayed H/M at ^123^I-MIBG scintigraphy [7].

Since semiquantitative analysis of myocardial ^123^I-MIBG uptake is characterized by a low interindividual and a within-subject variability [8], ^123^I-MIBG scintigraphy has become a valuable tool to provide information regarding the potential and actual benefit of pharmacological treatment in patients with HF [7].

Implantable cardioverter defibrillators (ICDs) and cardiac resynchronization therapy (CRT) are also useful therapeutic options in patients with HF. ICDs are dedicated to pacing and/or defibrillating lethal cardiac arrhythmias, whereas CRT improves the electrical dyssynchrony found in many patients with HF and thereby can improve mechanical dyssynchrony leading to increased left ventricular function.

The purpose of this paper is to review published scientific data regarding the clinical usefulness of ^123^I-MIBG scintigraphy in HF patients referred for ICD or CRT.

## 2. Role of ^123^I-MIBG Scintigraphy in HF Prognosis

Several studies have shown that the assessment of the cardiac autonomic state with ^123^I-MIBG scintigraphy can help to estimate the prognosis and to monitor the effects of therapeutic interventions in HF patients [9–14].

First, Merlet et al. [9] demonstrated that impaired cardiac sympathetic innervation, as assessed with ^123^I-MIBG imaging, was associated with adverse cardiac outcomes in 112 patients with HF and a reduced left ventricular ejection fraction (LVEF). After a mean followup of 27 ± 20 months, the only independent predictors for mortality were low myocardial ^123^I-MIBG uptake (*P* < 0.01) and LVEF (*P* = 0.02) [9].

Then, Agostini et al. [10] enrolling 290 HF patients from six centers reported that the mean H/M at ^123^I-MIBG scintigraphy was 1.51 ± 0.30 in patients who had major cardiac events (MCEs) and 1.97 ± 0.54 in patients without MCEs (*P* < 0.001). When an optimum H/M was set at 1.75, two-year event-free survival was 62% for H/M under this threshold and 95% for H/M greater than or equal to 1.75 (*P* < 0.0001). Moreover, H/M and LVEF were the only significant predictors of MCEs. The authors confirmed the prognostic value of ^123^I-MIBG scintigraphy in HF patients, and its potential to identify a quantitative threshold that may help to estimate the risk of cardiac mortality and potentially fatal ventricular arrhythmias [10].

Recently, the ADMIRE-HF study [11] confirmed previous data. The investigators prospectively evaluated ^123^I-MIBG imaging for identifying symptomatic HF patients (NYHA class II/III and LVEF ≤35%) most likely to experience cardiac events. Among the 961 patients enrolled, 25% experienced events (median follow-up 17 months). The cardiac event risk (primary end point) was significantly lower for subjects with H/M ≥1.60. For H/M <1.60, 2-year probabilities of cardiac death and all-cause mortality were 11.2% and 16.1% versus 1.8% and 3.0% for the group with H/M ≥1.60. Significant contributors to the multivariable model were H/M, LVEF, brain natriuretic peptide (BNP), and NYHA functional class. ^123^I-MIBG imaging also provided additional discrimination in analyses of interactions between BNP, LVEF, and H/M. Moreover, 9% of the patients experienced either nonfatal arrhythmic events (self-limited ventricular tachycardia, *n* = 12; resuscitated cardiac arrest, *n* = 6; appropriate ICD discharges, *n* = 45) or sudden cardiac death (*n* = 23). These combined “arrhythmic” events were significantly more common in subjects with H/M <1.60. ADMIRE-HF study provided prospective validation of the independent prognostic value of ^123^I-MIBG scintigraphy in the assessment of patients with HF [11].

Some clinical trials have demonstrated that ^123^I-MIBG scintigraphy parameters are predictive of sudden cardiac death in patients with mild-to-moderate HF independently of LVEF [12, 13]. Kasama et al. showed that delta WR obtained from serial 123I-MIBG scintigraphic studies was a useful parameter for predicting cardiac death and sudden death in stabilized patients with HF [12]. Moreover, Tamaki et al. found that cardiac MIBG WR, but not signal-averaged electrocardiogram (SAECG), heart rate variability (HRV), or QT dispersion, was a powerful predictor of sudden cardiac death in patients with mild-to-moderate HF, independently of LVEF [13]. Bax et al. [14] examined the relationship between abnormalities of ventricular sympathetic innervation delineated by scintigraphic imaging with ^123^I-MIBG and inducible ventricular tachyarrhythmias in patients with left ventricular dysfunction and previous myocardial infarction. These authors demonstrated that ^123^I-MIBG scintigraphy abnormalities are associated with lethal arrhythmias and may guide appropriate ICD implantation in patients affected by HF [14].

## 3. Cardiac ^123^I-MIBG Imaging in HF Patients with ICD

One of the first studies performed to explore any role of ^123^I-MIBG scintigraphy in patients with ICD was designed by Rigden et al. who demonstrated that ICD shocks did not cause any change in sympathetic nerve activity in 11 patients who underwent ^123^I-MIBG scans before and after ICD implantation [15].

Although the ICD has been shown to abort potentially fatal ventricular arrhythmias, identification of patients who will most benefit from this device remains difficult. Despite widespread use of ICDs, their cost and the fact that only a certain group of patients fully benefits from the devices require appropriate risk stratification of patients. In the last years several studies were designed in order to identify patients who will benefit most from an ICD (Table 1).

Arora et al. [16] undertook a pilot study to evaluate myocardial sympathetic innervation with the use of ^123^I-MIBG imaging, as well as central autonomic tone with the use of heart rate variability (HRV) analysis, in patients with ICD. Test results were correlated with the occurrence of ICD discharges. Seventeen patients with previously implanted defibrillators were studied. Of these, 10 had at least 1 appropriate device discharge for ventricular tachyarrhythmias, whereas 7 had no discharge. Patients with a discharge had a significantly lower ^123^I-MIBG H/M, more extensive sympathetic denervation, and significantly reduced values for several HRV parameters [16]. This was the first study to demonstrate that cardiac autonomic assessment using ^123^I-MIBG imaging may help to select patients who would most benefit from an ICD by identifying those at increased risk for potentially fatal arrhythmias.

Then, Nagahara et al. [17] investigated whether altered cardiac autonomic function is associated with the occurrence of ICD discharge or lethal cardiac events. Fifty-four ICD-treated patients were prospectively followed after assessment of cardiac ^123^I-MIBG uptake (quantified as H/M), plasma concentration of BNP, and LVEF. Patients were divided into 2 groups based on the presence (group A) or absence (group B) of appropriate ICD discharge and followed up for a 15-month period. Group A had a significantly lower level of cardiac ^123^I-MIBG uptake and a higher plasma BNP level than did group B. Univariate analysis revealed that plasmatic BNP levels, any medication, and late H/M were predictors of lethal cardiac events, whereas multivariate analysis showed that only late H/M was a significant predictor. Thus, the authors concluded that when combined with plasma BNP concentration or cardiac function, cardiac ^123^I-MIBG uptake is closely related to lethal cardiac events and can be used to identify patients who would benefit most from an ICD [17].

Koutelou et al. [18] studied 25 patients with a recent ICD and mild HF (NYHA I-II) due to either ischemic (*n* = 15) or dilated (*n* = 10) cardiomyopathy. One week after ICD implantation they underwent baroreflex sensitivity (BRS) evaluation, 24-h HRV assessment, and ^123^I-MIBG imaging. The mean patient followup was 32 ± 10 months. The frequency of fast ventricular arrhythmic episodes (FVAE) demonstrated a positive correlation to WR at ^123^I-MIBG imaging (*P* = 0.001). WR was also independent predictor of FVAE [18].

Nishisato et al. [19] quantified cardiac ^123^I-MIBG uptake in 60 patients with ICD who were prospectively followed with endpoints of appropriate ICD shocks or cardiac death. Cardiac ^123^I-MIBG was quantified as H/M. During a mean 29-month interval, ICD shock was documented in 30 patients (50%); three cardiac deaths were also observed in this group of patients. Patients with ICD shocks had a significantly smaller H/M than did those without ICD shocks [19].

Boogers et al. [20] evaluated whether ^123^I-MIBG imaging predicts ventricular arrhythmias causing appropriate ICD therapy (primary end point) and the composite of appropriate ICD therapy or cardiac death (secondary end point). One hundred sixteen HF patients referred for ICD therapy underwent ^123^I-MIBG scintigraphy and myocardial perfusion imaging before ICD implantation. During a mean followup of 23 ± 15 months, appropriate ICD therapy was documented in 24 (21%) patients and appropriate ICD therapy or cardiac death in 32 (28%) patients. Reduced myocardial ^123^I-MIBG uptake was an independent predictor for both end points [20].

## 4. Cardiac ^123^I-MIBG Imaging in HF Patients Undergoing CRT

Current guidelines recommend CRT in HF patients who remain symptomatic in NYHA classes III-IV despite optimal pharmacological therapy, with LVEF <35%, LV dilatation (LV end diastolic diameter >55 mm) normal synus rhythm and wide QRS complex (≥120 ms) [21].

Since CRT has been shown to reduce mortality due to reverse remodeling (reduction in ventricular volumes and improvement in systolic function), as well as to changes in sympathetic activity, some authors investigated whether CRT affects cardiac sympathetic nerve activity, and whether changes are related to CRT response [22–27] (Table 2).

Higuchi et al. [22] evaluating 18 HF patients observed that those who achieved cardiac resynchronization after biventricular pacing had a significant improvement in cardiac symptoms, exercise capacity, and cardiac sympathetic function, as estimated by the delayed H/M at ^123^I-MIBG imaging [22].

Nishioka et al. [23] studied 30 patients with HF and classic indications for CRT who were prospectively followed before and at least 3 months after CRT. After CRT, patients were divided into two groups: group 1 (21 patients), responders improving to functional class (FC) I or II, and group 2 (9 patients), nonresponders remaining in FC III or IV. After CRT, only group 1 showed favorable changes in QRS width (*P* = 0.003), LVEF (*P* = 0.01), and H/M at ^123^I-MIBG imaging (*P* = 0.003). The H/M ratio and WR were associated with CRT response (*P* = 0.005 and *P* = 0.04, resp.). Moreover, the H/M was the only independent predictor of CRT response (*P* = 0.01) [23].

A similar trial was performed by Burri et al. [24] who reported that CRT responders showed a significant decrease in WR at ^123^I-MIBG imaging at followup when compared with baseline (*P* = 0.036) and also had a significantly lower WR at followup when compared with nonresponders (*P* = 0.002) [24].

Cha et al. [25] demonstrated the H/M at ^123^I-MIBG imaging increased (1.82 versus 1.97; *P* = 0.03), whereas the WR was reduced (48% versus 37%; *P* = 0.01) in 45 consecutive patients with HF who received CRT. Compared with nonresponders, CRT responders had a higher delayed H/M ratio (2.11 versus 1.48; *P* = 0.003) and a lower WR (37% versus 62%; *P* = 0.003) at baseline [25].

Moreover, Tanaka et al. [26] found that HF patients with dyssynchrony showed less cardiac sympathetic activity than did those without dyssynchrony even though LVEF was not significantly different (24 ± 6% versus 25 ± 7%). Dyssynchrony and H/M at ^123^I-MIBG imaging ≥1.6 were associated with a high frequency of LV functional improvement with a higher response rate [26].

Lastly Shinohara et al. [27] confirmed previous data as they found that delayed H/M at ^123^I-MIBG imaging significantly increased in the CRT responders but not in the nonresponders [27].

## 5. General Remarks

Current indications for ICD implantation in primary prevention are referred to patients “with LV dysfunction due to prior myocardial infarction (MI) who are at least 40 days post MI, have LVEF less than or equal to 30% to 40%, are NYHA functional class II or III, are receiving optimal medical therapy and who have reasonable expectation of survival with a good functional status for more than one year.” Moreover, ICD is recommended for primary prevention in “patients with non-ischemic dilated cardiomyopathy who have a LVEF less than or equal to 30% to 35%, are NYHA functional class II or III, who are receiving chronic optimal therapy and who have reasonable expectation of survival with a good functional status for more than 1 year.” Despite the current criteria, many subjects still die because they were considered low-risk patients [28].

Conversely, many patients with an ICD device implanted after an abortive cardiac arrest or a life threatening ventricular tachycardia never experience appropriate ICD shock, and they are also exposed to unnecessary ICD shocks, device malfunction or infection, and higher risk of anxiety or depression. 

Moreover, there are some cardiac diseases without left ventricular dysfunction which are associated with high risk of lethal arrhythmyas (such as arrhythmogenic right ventricular cardiomyopathy, long QT syndrome, and Brugada syndrome). ^123^I-MIBG scintigraphy compared to other imaging modalities is the only able to evaluate the sympathetic cardiac innervations status in these settings and help to stratify patients who will most benefit from ICD implantation for primary prevention [28].

Heart rate variability is noted as the most sensitive marker of autonomic dysfunction; however, it seems, to be influenced by artifacts, rhythm disturbance, and noncardiac autonomic tone that makes it not reliable for stratifying HF patients.

^123^I-MIBG scintigraphy is emerging as a powerful tool to assess prognosis in patients with HF and as an effective guide to evaluate appropriate ICD implantation in patients at high risk of sudden cardiac death. Most of the studies evaluated global cardiac ^123^I-MIBG activity, but regional SPET analysis is possible. However, it is difficult to reconstruct SPET data when global ^123^I-MIBG uptake is severely reduced; also, it is well known that MIBG uptake is underestimated at inferior and apical wall probably for attenuation or physiologic reason.

Fewer data are available from scientific literature, and further clinical trials are needed to investigate the potential role of ^123^I-MIBG imaging for better selection of ICD candidates.

It is well known that biventricular pacing reduces ventricular dyssynchrony and improves LVEF. Moreover, several studies demonstrated that CRT improved cardiac sympathetic activity assessed by ^123^I-MIBG scintigraphy. Available data showed that CRT responders showed a rebalance in cardiac autonomic function as structural reverse remodeling may also be accompanied by reverse modulation of the cardiac sympathetic system, with attenuation of the excessive sympathetic drive—associated with HF—and with rebalance of cardiac autonomic activity. For these reasons, ^123^I-MIBG scintigraphy may have a potential role in predicting response to biventricular pacing. However, all these cited studies have a small sample size of patients. Further larger clinical trials are needed to understand whether cardiac ^123^I-MIBG scintigraphy may have a role in selecting HF patients who will better respond to CRT.

Another issue that needs to be further addressed is the heterogeneity of data acquisition and image analyses about ^123^I-MIBG scintigraphy among different studies. Some efforts have been done to standardize this technique (like the optimal collimator to use) and to contribute to its clinical implementation for prognostication of HF patients undergoing ICD and CRT [29–31].

## 6. Conclusion

^123^I-MIBG scintigraphy seems to be a useful tool in cardiac dysfunction to assess which patients may benefit most from an ICD implantation to reduce the risk of ventricular arrhythmia or sudden cardiac death. Moreover, ^123^I-MIBG scintigraphy seems to predict which HF patients will respond to CRT with an improvement in left ventricular function.

## Figures and Tables

**Table 1:** Studies about the usefulness of ^123^I-MIBG scintigraphy in planning ICD.

Authors	Country	Number of patients	MIBG parameters evaluated
Rigden et al. [15]	USA	11	MIBG uptake
Arora et al. [16]	Japan	17	Early and late H/M ratio; WR; early and late MIBG defect score
Nagahara et al. [17]	Japan	44	Early and late H/M ratio; WR
Koutelou et al. [18]	Greece	25	Early and late H/M ratio; WR
Nishisato et al. [19]	Japan	60	Early and late H/M ratio; WR
Boogers et al. [20]	Nederland	116	Early and late H/M ratio; WR; early and late MIBG defect score

H/M: heart to mediastinum ratio; WR: washout rate.

**Table 2:** Studies about the usefulness of ^123^I-MIBG scintigraphy in patients who underwent CRT.

Authors	Country	Number of Patients	MIBG parameters evaluated
Higuchi et al. [22]	Japan	18	Early and late H/M ratio; WR; early and late MIBG defect score
Nishioka et al. [23]	Brazil	30	Early and late H/M ratio; WR
Burri et al. [24]	Nederland	16	Early and late H/M ratio; WR
Cha et al. [25]	USA	45	Late H/M ratio; WR
Tanaka et al. [26]	Japan	50	Early and late H/M ratio; WR
Shinohara et al. [27]	Japan	27	Late H/M ratio

H/M: heart to mediastinum ratio; WR: washout rate.
